# Mice Hemizygous for a Pathogenic Mitofusin-2 Allele Exhibit Hind Limb/Foot Gait Deficits and Phenotypic Perturbations in Nerve and Muscle

**DOI:** 10.1371/journal.pone.0167573

**Published:** 2016-12-01

**Authors:** Peter Bannerman, Travis Burns, Jie Xu, Laird Miers, David Pleasure

**Affiliations:** 1 Institute for Pediatric Regenerative Medicine, Shriners Hospitals for Children, Northern California, Sacramento, California, United States of America; 2 Department of Cell Biology and Human Anatomy, University of California Davis, Davis, California, United States of America; 3 Department of Neurology, University of California Davis, Sacramento, California, United States of America; University of Sydney, AUSTRALIA

## Abstract

Charcot-Marie-Tooth disease type 2A (CMT2A), the most common axonal form of hereditary sensory motor neuropathy, is caused by mutations of mitofusin-2 (MFN2). Mitofusin-2 is a GTPase required for fusion of mitochondrial outer membranes, repair of damaged mitochondria, efficient mitochondrial energetics, regulation of mitochondrial-endoplasmic reticulum calcium coupling and axonal transport of mitochondria. We knocked T105M MFN2 preceded by a loxP-flanked STOP sequence into the mouse Rosa26 locus to permit cell type-specific expression of this pathogenic allele. Crossing these mice with nestin-Cre transgenic mice elicited T105M MFN2 expression in neuroectoderm, and resulted in diminished numbers of mitochondria in peripheral nerve axons, an alteration in skeletal muscle fiber type distribution, and a gait abnormality.

## Introduction

CMT2A, the most common axonal form of Charcot-Marie-Tooth disease, is caused by mitofusin-2 (MFN2) mutations [1–5]. CMT2A nerve biopsy specimens reveal axon loss, in some cases accompanied by abnormal mitochondrial groupings and morphologies in Schwann cells and axons [2,6,7]. Toxic gain-of-function, rather than haploinsufficiency, may be responsible for dominant inheritance in CMT2A [8–9]. In support of this hypothesis, deletion of one MFN2 allele in mice is asymptomatic, as are truncating MFN2 mutations in humans, unless inherited in conjunction with an additional mutation in MFN2 or another CMT-associated allele [10–11].

MFN2 is a dynamin-like trans-membrane GTPase that, in a complex with MFN1, catalyzes mitochondrial tethering and outer membrane fusion [12–15]. MFN2 also couples mitochondria to endoplasmic reticulum [16]. Homozygous MFN2 knockout blocks fusion-induced mitochondrial repair, depletes mitochondrial DNA, impairs mitochondrial respiration and Ca^2+^ homeostasis, prevents mitophagy of damaged mitochondria, and increases vulnerability of cultured cells to apoptosis [12,14,17–22]. In neurons, MFN2 participates in Miro/Milton-mediated tethering of mitochondria to the kinesin complex [23], and MFN2 deficiency or expression of pathogenic MFN2 blocks axonal mitochondrial transport and leads to axonal degeneration [24–27].

**Fig 1:**
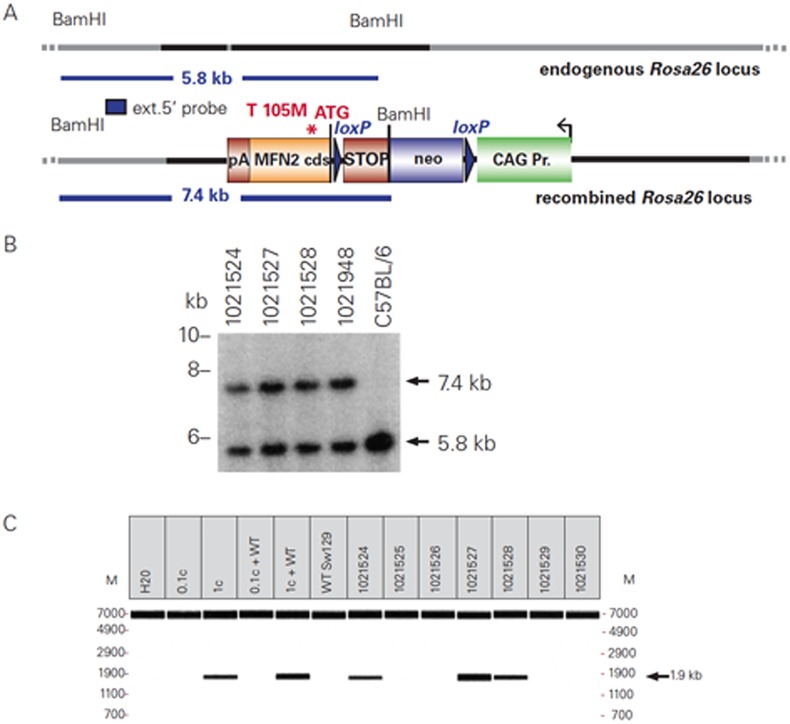
STOP-MFN2^T105M^ construct and its’ insertion into the Rosa26 locus of C57BL/6 mice. Panel A shows genomic DNA of tested F1 generation mice was compared with wild-type C57BL/6 mouse DNA by Southern blot analysis in Panel B, showing that, amongst the 4 hemizygous F1 mice illustrated in this panel, all tested positive for the Rosa26 recombined allele. PCR genotyping of mice derived from the breeding of chimeras with wild-type C57BL/6 mice are shown in Panel C.

Availability of an in vivo mouse model that mirrors human CMT2A would facilitate genetic analysis of CMT2A pathogenesis and pre-translational testing of CMT2A therapies. However, most of the currently available CMT2A models, which employ a neuron-specific promoter (HB9 or neuron-specific enolase) to drive expression of a mutant MFN2 transgene, elicit neuropathy only when the transgene is inherited from both parents (e.g.[28]), whereas CMT2A is almost always dominantly inherited; this discrepancy in inheritance pattern suggests that the pathogenesis of neuropathy in these mouse models may differ from the presumptively dominant negative mechanism responsible for the human disorder. However, a recent study characterizing deficits associated with the Arg94Trp (R94W) knock-in mutation did find that these mutant mice exhibited decreased mobility in open-field testing but not other gaiting parameters, as well as a small but statistically relevant decrease in axon size and myelination in mice hemizygous for this mutation [29]. Another deficiency of currently available CMT2A models is that, in contrast to human CMT2A, expression of the pathogenic MFN2 allele in these mouse models is, by design, limited to neurons, therefore not permitting analysis of the role, if any, of other cell types including skeletal muscle expression of the mutant allele in the genesis of CMT2A phenotype. This may be a significant limitation, in view of accumulating evidence that mutant gene expression in other cell types does influence the progression of familial mutant SOD1 ALS and other neurodegenerative disorders. We chose to focus on an MFN2 knock-in mutation that is prevalent in the USA, namely Thr105Met (T105M) [30–31]. To ensure that this mutation causes a clinical phenotype in mice, we ‘knocked in’ human MFN2^T105M^ preceded by a STOP sequence, into the ubiquitous mouse Rosa26 locus [32]. Details of the construct, and validation of the mutant mice, are shown in Fig 1. To test the effects of systemic (neural + non-neural) excision of the STOP sequence from these mice, we crossed them to mice carrying a widely expressed, tamoxifen-inducible CAG-Cre-ER^T2^ transgene, a strong, highly efficient promoter element [33]. Once we established that this produced a major phenotype involving abnormal mitochondrial distribution as predicted by many previous studies in the homozygous mice, we then bred out the tamoxifen inducible CAG promoter and crossed these mice to nestin-cre mice to limit the expression of MFN2 to cells of neuroectodermal derivation i.e. PNS and CNS neurons and neuroglia as well as certain non-neural cells such as skeletal muscle satellite cells in the hemizygous condition. To investigate muscle/nerve interactions in hemizygous T105M MFN2 nestin-cre mice, we studied the lower hind limb musculature including the soleus and peroneus longus (fibularis longus) muscles, the former of which plays a major role in plantarflexion of the feet, as well as the lateral gastrocnemius, which plays a primary role in calf flexion during fast running and a lesser role in plantarflexion. We also analyzed the tibialis anterior, which mediates dorsiflexion actions in the feet, thereby acting as an antagonist muscle to the soleus. Using the above hemizygous T105M MFN2 nestin-cre mice in combination with the sensitive Noldus Catwalk system we identified a phenotypic model of human pes cavus.

To investigate perturbations in hind limb peripheral nerves, we focused on the tibial nerve whose axons innervate the soleus and gastrocnemius muscle as well as distal muscles in the foot including the flexus digitorium brevis and flexor hallucis brevis muscles [34].

## Methods

### Construction of the Rosa-STOP-MFN2^T106M^ mice and nestin-cre-MFN2^T106M^ mutant mice

We knocked in human MFN2^T105M^, preceded by a STOP sequence, into the ubiquitous mouse Rosa26 locus [32]. Details of the construct, and validation of the mutant mice, are shown in Fig 1.

To test the effects of systemic (neural + non-neural) excision of the STOP sequence from these mice, we crossed them to mice carrying a widely expressed, tamoxifen-inducible CAG-Cre-ER^T2^ transgene [33]. We determined that these mice developed a major diseased phenotype beginning 4–6 weeks following tamoxifen treatment (dosed at two months of age with1mg/day IP for 5 days) versus tamoxifen treated wild-type C57Bl/6 control mice. We have used this dosing regime in previous fate-mapping studies using ROSA-EYFP mice without potential toxic side-effects of tamoxifen treatment [35–36].

The diseased phenotype appeared to result from multiple organ perturbations including respiratory distress, skin follicle erection, bloating due to ascites accumulation and loss of mobility. Since these animals were not fit to perform any gaiting measurements relative to their normal tamoxifen treated controls the animals were either processed for immunohistochemical analysis or humanely euthanized by CO2 inhalation. Once we had determined that these mice developed such a major diseased phenotype we then tested the hypothesis that hemizygous expression of MFN2^T106M^ induced a phenotype with hind limb dysfunction in a restricted subset of cells including peripheral neurons, Schwann and muscle satellite cells. To this end, we bred out the tamoxifen inducible CAG-cre motif and replaced it with the nestin-cre driven promoter obtained from JAX mice (JAX stock # 003771). Male mice between 10–12 weeks of age were used throughout these studies.

### Rotarod testing

Measuring accelerating rotarod retention times tested motor function. Mice were exposed to a rotarod starting speed of 4 rotations/minute (rpm), with subsequent increases in speed step rotation of 1.3 rpm every 10 seconds. The mice underwent daily 5-minute training sessions for 10 days until they had attained plateau performances to use as baseline retention times for statistical analysis with n = 6 for mutant and control mice.

### Noldus Catwalk

The gaits of hemizygous nestin-cre-MFN2^T106M^ mutant and control nestin-cre mice were measured using the Noldus Catwalk system. A minimum of three compliant runs (< 6 seconds in duration) were acquired and then classified using Noldus Catwalk XT 9.1 software. From the statistics generated, we chose to compare measurements of forward, intermediate toe spread and print length with n = 9 per strain.

### Tissue processing and immunohistology

Control MFN2 mutant mice were deeply anesthetized with ketamine (150mg/kg) and xylazine (16mg/kg), then transcardially perfused with phosphate buffered saline (PBS) followed by 4% paraformaldehyde (EM Sciences, Hatfield, PA) in PBS. Brain, spinal cord, kidney, liver and tibial nerve were removed first. To obtain transverse sections approximating the midline of entire muscle groups, the calf was cut below the knee and above the ankle joints. Tendons were teased away from the tibia and fibula prior to removal of both bones at their point of fusion above the ankle joint. Muscle tissue was analyzed from the right hand side of 3 separate mice of each strain. All tissue was post-fixed in fresh fixative for a further 1–2 hours (h) at 4°C. Samples were cryoprotected in 30% sucrose in PBS for 48–72 h at 4°C prior to embedding in OCT mounting medium and frozen in cryomolds with liquid nitrogen cooled 2-methylbutane. In the case of calf muscle tissue, the distal end was grasped with forceps and was initially partially frozen in OCT and 4mm of muscle tissue was cut away from the proximal end of the calf muscles using a single edged razor blade before mounting the new cleanly cut transverse muscles proximal end down in the base of the cryomold prior to freezing in OCT. Ten micron cryosections were cut using a Leica cryostat (model CM1950) and collected on glass slides. Table 1 provides details of the primary antibodies used in this study.

**Table 1:** Primary Antibodies Used for Immunohistology.

Antigen	Antibody
Slow myosin ATPase	Mouse monoclonal anti-slow Myosin, AbCam 1:100
Fast myosin ATPase	Rabbit polyclonal anti-fast Myosin, AbCam 1:100
Collagen type IV	Goat anti-collagen IV
MAP2a	Chicken polyclonal, Millipore 1:500
Mouse specific mfn2	Rabbit polyclonal, Epitomics 1:100
Human speciic mfn2	Rabbit polyclonal Cell Signalling
Sarcomeric actin	Rabbit polyclonal, Sigma, 1:500
VDAC	Rabbit poyclonal, Abcam
Myelin Basic Protein	Rat anti-MBP, gift of V.Lee, U Penn,1:2
NeuN	Mouse anti-NeuN, Millipore, 1:500
Synaptophysin	Rabbit monoclonal anti-Synaptophysin,Millipore, 1:200

Fluorescent conjugated secondary antibodies (species specific) were from Jackson ImmunoResearch, and were used at 1:500 or 1:1000.

### Muscle immunolabelling

Slides were pre-incubated in blocking solution composed of either 10% donkey or goat serum diluted in minimum essential medium containing 15 mM HEPES and 0.05% sodium azide for 1h. This blocking solution was also used as a diluent for both primary and secondary antibody incubations. Sections were incubated with primary antibodies overnight at 4°C and secondary antibodies for 30min at room temperature with three 5 min PBS washes between each step.

When a mouse monoclonal antibody was used as a primary antibody (e.g. mouse anti-slow myosin ATPase), tissue sections were pretreated with donkey or goat anti-mouse Fab fragments (260 μg/ml, Jackson Immunoresearch Labs, West Grove, PA) to block binding to endogenous mouse immunoglobulins. Detection of primary antibody binding was performed using species-specific fluorochrome-conjugated second antibodies (Jackson Immunoresearch). When the biotin/streptavidin secondary detection system was used to enhance the sensitivity of immunofluoresence detection, sections were also pre-treated with the biotin/streptavidin blocking kit as per the manufacturer’s instructions (Vector Labs). Following secondary detection, cryostat sections were post-fixed with cold methanol (-20°C) and counterstained with DAPI.

### Confocal microscopy

Immunolabeled sections were imaged using a Nikon upright microscope (Nikon Eclipse 90i) coupled to an A1 laser scanning confocal head with a multi-laser launch coupled through a single mode fiber. Confocal images were processed using Nikon NIS-Elements software. The diameters of individual muscle fibers were obtained by measuring the lesser diameter of each fiber. The diameters of 50–70 muscle fibers were counted from 4 control and heterozygote MFN2 mutant mice. To obtain these counts, typically, a central field in each muscle mass was established, then fields above, lateral and below were captured for counting. Since slow fibers comprise a minor subpopulation of fibers compared to fast fibers in the peroneus longus (Peron), gastrocnemius (Gast) and tibialis anterior (Tib), therefore only fast fibers were counted in these muscles. In contrast, the soleus (Sol) muscle contains many slow and fast fibers and hence both types of muscle fiber were counted in this muscle.

### Transmission electron microscopy of tibial nerves

Control and MFN2^T105M^ mutant mice were deeply anesthetized with ketamine/xylazine (as above), then transcardially perfused briefly with 500 units of heparin in 0.1M Sorensens’s phosphate buffer (pH 7.4) followed sequentially with freshly prepared 4% paraformaldehyde then 3% glutaraldehyde all in 0.1M phosphate buffer (pH 7.4). A segment of tibial nerve (2-3mm in length) was excised 3mm distal to the point of separation from the main branch point of the sciatic nerve and immersion fixed for a further 2 days in 3% glutaradehyde at 4°C. Tissue was washed with 0.2M sodium cacodylate buffer (pH 7.2) then post-fixed with 2% aqueous osmium tetroxide for 2 hr. Samples were washed with cacodylate buffer then dehydrated through ascending alcohols, washed with propylene oxide and embedded in EMBed-812 resin (Electron Microscopy Sciences, Hatfield, PA). For light microscopy, one micron semi-sections were cut on a Leica EMU6 microtome and counterstained with toluidine blue. For electron microscopy, ultrathin sections (70-80nm) were cut on a Leica EM UC7 microtome and collected on 1x2mm formvar coated copper slot grids. Sections were double stained with uranyl acetate and lead citrate and examined on a Philips CM120 electron microscope.

To quantify the number of mitochondria per axon, the numbers of transected mitochondrial profiles were quantified in 600–750 axons per tibial nerve. For calculating the mean axonal diameter and g-ratios of tibial nerve axons, at least 120 axons were counted per tibial nerve. The above TEM studies were performed on right hand side tibial nerves from 3 separate mice of each strain.

Ethics Statement: All animal experimentation was performed in accordance with UCDavis IACUC protocol #18135, approved 06/15/2015

## Results

### Disrupted phenotype and mitochondrial aggregation in hemizygous Rosa-STOP-MFN2^T105M^/CAG-CreER^T2^

These mice exhibited severe distress with multiple organ failure as detailed above, within 6 weeks after completion of the course of tamoxifen. Those mice not harvested for immunohistochemical studies soon after they became symptomatic were euthanized for humane reasons. Since this phenotype was drastically different compared to normal healthy tamoxifen treatment control mice (and the human disease), there was no value in performing rotarod analysis.

**Fig 2:**
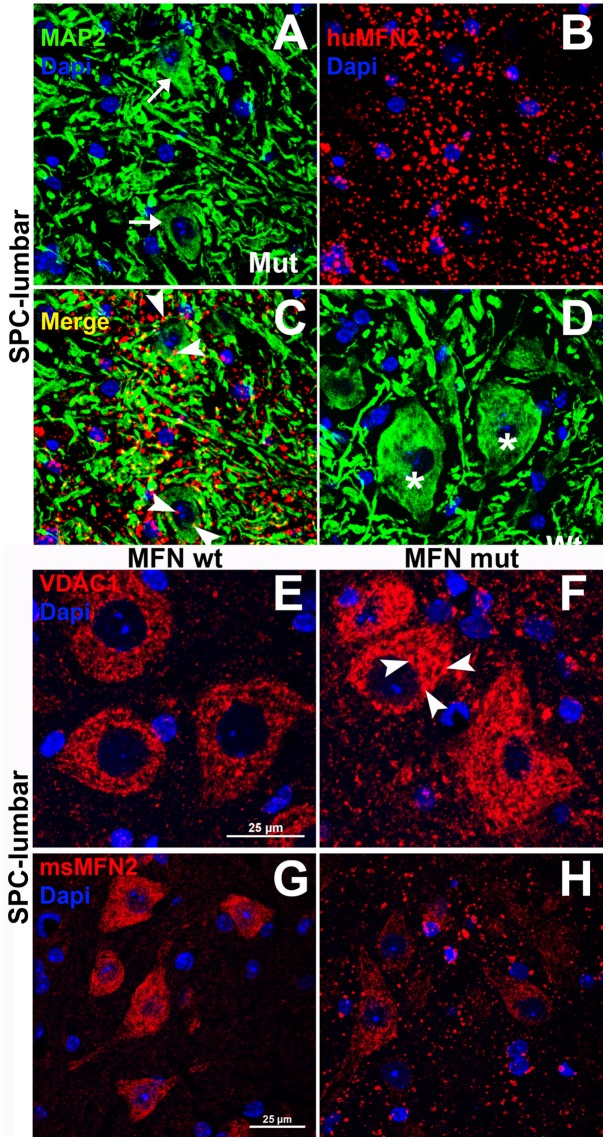
Dual label immunofluorescence indicating mitochondrial aggregation in MFN2 mutant versus control mice. Mitochondrial immunolabelling using antibodies specific to human MFN2 in the ventral horn of the spinal cord (SPC). Sections were co-stained with ant-MAP2 to identify the perikarya and dendrites in wild-type and MFN2 mutant mice. Figures A, B and C show an identical field labeled with antibodies to MAP2 (A) and human MFN2 (B) with figure C showing the merged images of A and B. Figure D shows the absence of human MAP2 immunolabelling in wild-type tissue. Note the extensive ‘clumped’ pattern of anti-human MFN2 immunolabelling throughout the neuropil in astrocytes/oligodendrocytes, including aggregates (arrowheads in C) in neuronal perikarya (arrows in A). Micrographs E and F demonstrate mitochondrial immunolabelling using antibodies to VDAC (red) in wild-type (E) and mutant spinal cord (F) in which mitochondrial clumping is evident (arrowheads), in line with the results in A-C. Micrographs G and H represent mitochondrial immunolabelling using antibodies specific to mouse MFN2 in wild-type (G) and mutant ventral spinal cord (H). Note the robust immunolabelling in G but relative paucity in H. Mutant tissue was obtained from 4-week post-tamoxifen Rosa-STOP-MFN2^T106M^/CAG-Cre-ER^T2^ hemizygous mice. All scale bars = 25μm.

Immunostaining with a rabbit monoclonal antibody that recognizes human MFN2 but not mouse MFN2 (Cell Signaling Ab 11925S), demonstrated expression of mutant human MFN2 in the ventral horn of the spinal cord (Fig 2A–2C) but no expression in control wild-type tissue (Fig 2D). Interestingly, when immunolabelling was performed using antibodies specific to endogenous mouse MFN2, wild-type tissue, as expected, expression was strong in wild-type tissue (Fig 2G) in line with mitochondrial immunodetection using antibodies to VDAC (Fig 2E) but unexpectedly low levels of immunostaining in corresponding mutant tissue (Fig 2H). The latter result is important in that it indicates that endogenous MFN2 expression levels are low and therefore unlikely to induce potential MFN2 toxicity, compared to a scenario where both endogenous and mutant forms of MFN2 were over expressed; although paradoxically a previous study has been shown that over-expression of wild-type MFN2 is neuroprotective both in vitro and in vivo [37].

### Disrupted phenotype and mitochondrial aggregation in non-neural tissue in hemizygous Rosa-STOP-MFN2^T105M^/CAG-CreER^T2^

**Fig 3:**
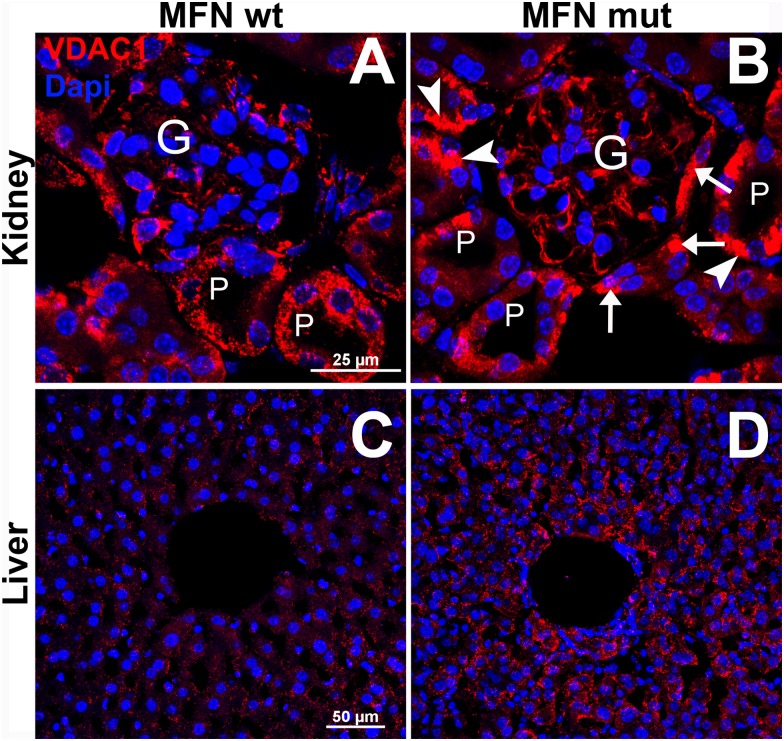
Mitochondrial VDAC immunolabelling in the renal cortex and liver parenchyma of wild-type and mutant mice. Figs A and B show mitochondrial immunodetection in wild-type and mutant tissue respectively. Fig 3C and D show corresponding immunolabelling in the liver parenchyma, each with a sinusoid in the center of the field. Comparing micrographs A and B within the cortical region of the kidney, note 1, the almost continuous red cytoplasmic within endothelial cells within the glomerulus (marked G), 2, more highly condensed VDAC immunolabelling in the epithelial cells of the Bowmans capusule (arrows) and epithelial cells of the proximal tubule (P, arrowheads) in the mutant versus wild-type tissue. Comparing micrographs C and D, note the more intense and clumped immunolabelling of mitochondria in hepatocytes surrounding a sinusoid in the mutant versus wild-type liver. Scale bar in A and B = 25μm and 50μm in C and D.

Fig 3 shows control mitochondrial VDAC immunostaining in wild-type kidney (A) and liver (C) tissue versus clumped/aggregated labeling in the corresponding mutant tissue (B and). These results may explain the multi-systemic failure of different organs in the hemizygous Rosa-STOP-MFN2T^105M^/CAG-CreER^T2^ mice.

**Fig 4:**
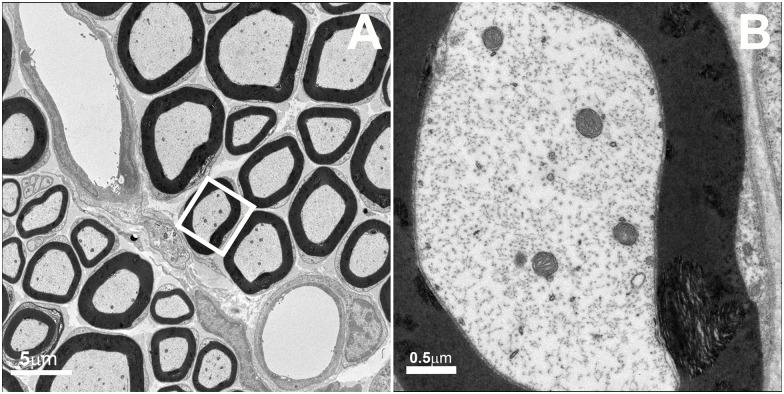
Transmission electron microscopy (TEM) of wild-type tibial nerve. Myelin sheaths and intra-axonal mitochondria appear normal. No Schwann cell mitochondria clusters are present. Size bar 5μm, Panel A, and 0.5μm, Panel B.

**Fig 5:**
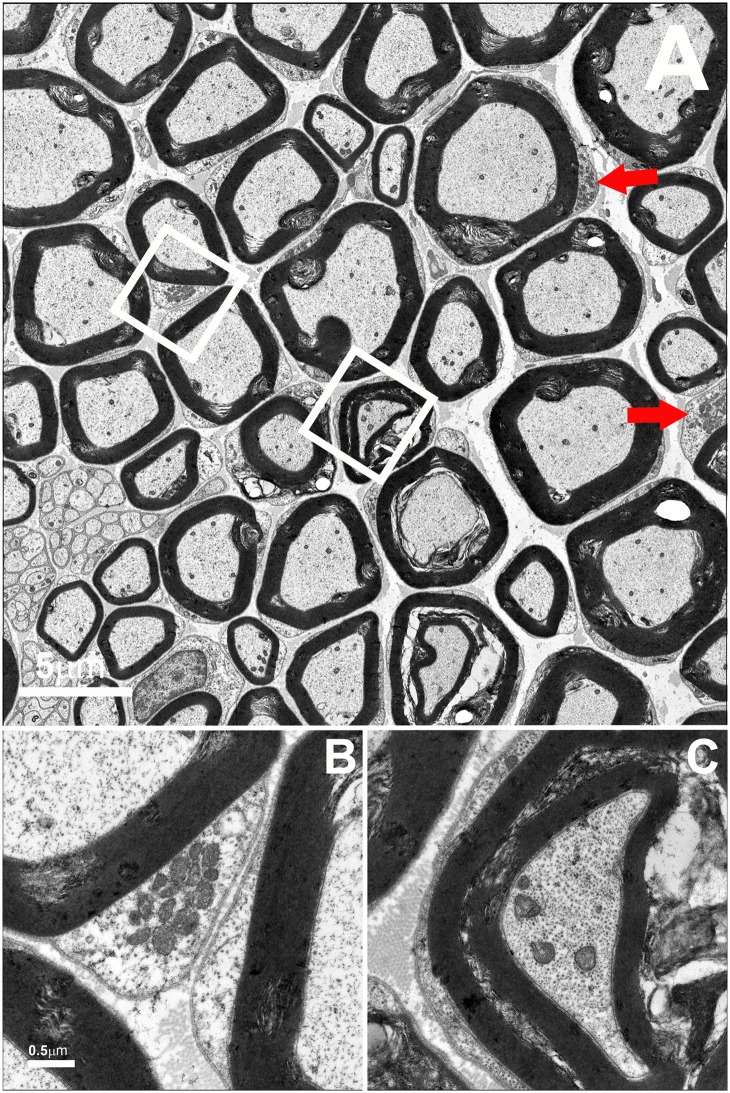
TEM of sciatic nerve from a Rosa-STOP-MFN2^T106M^/CAG-CreER^T2^ mouse. Micrographs show frequent clusters of mitochondria within Schwann cells (Box B and red arrows in upper panel low power view) similar to those previously described in CMT2A nerve biopsies [7]. Size-bars 5μm (large panel) and 0.5μm (small panels).

Compared to normal tibial nerve (Fig 4), TEM analysis of the tibial nerve of these mutant mice demonstrated both abnormal myelination and aggregation of small mitochondria in Schwann cell cytoplasm strongly suggested impaired fusion of mitochondria (Fig 5). Abnormal myelin configurations (see Panel C) surrounded some axons. Such profiles typically consisted of double myelinated axons in which the inner portion of the outer sheath was seen to be decompacting. While such profiles were occasionally present in normal tibial nerves, they were four times more frequent in mutant nerves (3.28 ± 0.09% profiles, mean ± SD, in control nerves versus 14.7 ± 1.08% profiles in mutant nerves, p< 0.001).

### Expression of mutant human MFN2 in spinal cord SPC ventral horn motor neurons of heterozygote nestin-cre-STOP-MFN2^T105M^

**Fig 6:**
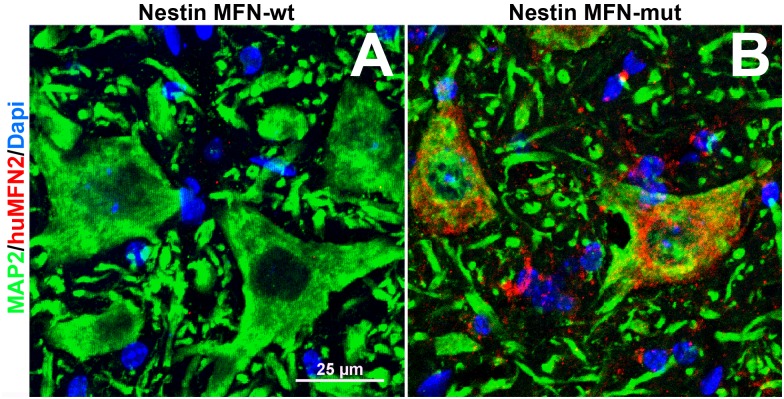
Expression of mutated human MNF2 in nestin MFN-mutant mice. 0.5μm confocal optical slice of transverse frozen sections of lumbar spinal cord showing anterior (motor) ventral horn neuronal cell bodies immunolabeled with anti-MAP2 (green) and anti-human MFN2 specific antibodies (red) in (A) nestin-cre control and B) nestin-Cre MFN-2 mutant mice. Note the normal diffuse distribution of mitochondria in plate B versus plate E in Fig 2. Non-neuronal cells also expressed mutant MFN2 (arrows) in the grey matter that were predominantly GFAP+ astrocytes. (Scale bar = 25 μm).

Fig 6 shows nestin-cre-driven expression of mutant form of human MFN2^T106M^ in mutant ventral horn neurons but not wild-type spinal cord. Astrocytes and oligodendroglia also expressed mutant MFN2 in-line with their neuroectoderm origin. Unlike in Fig 2, no aggregation/clumping of mitochondria was seen in neuronal perikarya of nestin-cre-STOP-MFN2^T105M^ motor neurons.

### Noldus Catwalk and abnormal print length in hemizygous nestin-cre-MFN2^T106M^ mutant mice

**Fig 7:**
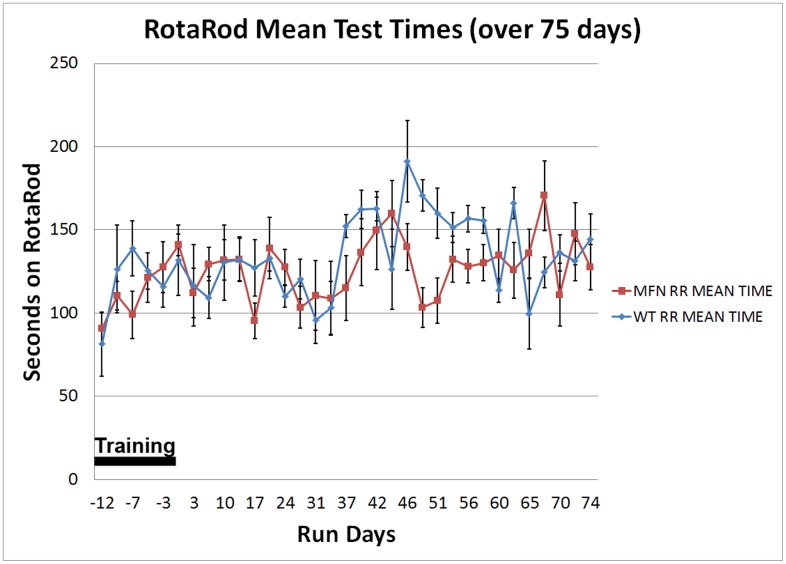
Longitudinal rotarod analysis of nestin-cre versus nestin-cre-MFN2^T106M^ mutant mice. The graphs show a longitudinal study, quantifying rotarod performance (secs) over a period of 75 days exclusive of an initial 12 day training period on the apparatus. This study was initiated when both nestin-cre control and nestin-cre-MFN2T105M mice were 10 weeks of age. The graph indicates no long-term difference between rotarod scores between control mice (n = 5) and test mice (n = 6). Vertical scale bars indicate SEMs.

For a summary of all the following data see Table 2. We did not detect measurable motor deficits based on simple inspection or accelerating rotarod analysis in these mice (Fig 7). However, using the Noldus Catwalk system, we analyzed 3 standard hind-foot metrics in control versus heterozygote MFN2 mutant mice including complete and intermediate hind-foot toe spread and total print length. While we did not detect differences in toe spread metrics, we did however detect a statistically significant decrease in print length of MFN2 mutant mice namely 0.58 ± 0.038 cm (mean ± standard deviation) versus wild-type littermate controls (0.71 ± 0.072 cm, p <0.0003, in 2-sample t-test, n = 9), a result reminiscent of the pes cavus foot deformity that occurs in patients with CMT2A2. The use of Noldus catwalk versus that of rotarod analysis exemplifies the use of the former to quantify subtle differences in gaiting parameters.

**Table 2:** Study Summary.

**Assay**	**# of Mice**	**Values (mean** ± **SD)**	**Statistical Relevance**
RotaRod Times	n = 6 mice	See Fig 6	NS
CatWalk/Print Length	n = 9 mice	Control = 0.71±0.072cm Mutant = 0.58±0.038cm	**Yes: p<0.0003**
Mitochondria per Tibial Axon	n = 3 tibial nerves	Control = 2.67±0.24 Mutant = 1.84±0.40	**Yes: p<0.012**
# of Myelinated Axons/Tibial Nerve	n = 3 tibial nerves	Control = 2299±154 Mutant = 2217±56	NS: p>0.05
Mean Axonal Diameter of Myelinated Axons in Tibial Nerve	n = 3 tibial nerves	Control = 3.63±0.25μm Mutant = 3.56±0.011μm	NS: p>0.05
G-ratio of Axons in Tibial Nerve	n = 3 tibial nerves	Control = 0.658±0.011 Mutant = 0.662±0.013	NS: p>0.05
**Muscle Fiber Diameter**	**# of Mice and Fiber Type**		
Peroneus	n = 4, Fast	Control = 31.5±3.3μm Mutant = 30.0±0.9μm	NS: p>0.05
Gastrocnemius	n = 4, Fast	Control = 38.8±2.9μm Mutant = 35.5±3.0μm	NS: p>0.05
Tibialis	n = 4, Fast	Control = 34.2±2.8μm Mutant = 30.7±1.0μm	**Yes: p<0.03**
Soleus	n = 4, Fast	Control = 30.0±1.8μm Mutant = 26.6±2.4μm	NS: p>0.05
Soleus	n = 4, Slow/Mixed	Control = 33.7±0.7μm Mutant = 30.0±0.6μm	**Yes: p<0.015**

NS = not significant

### Tibial nerves in MFN2 heterozygote mice contain fewer mitochondria compared to control mice

**Fig 8:**
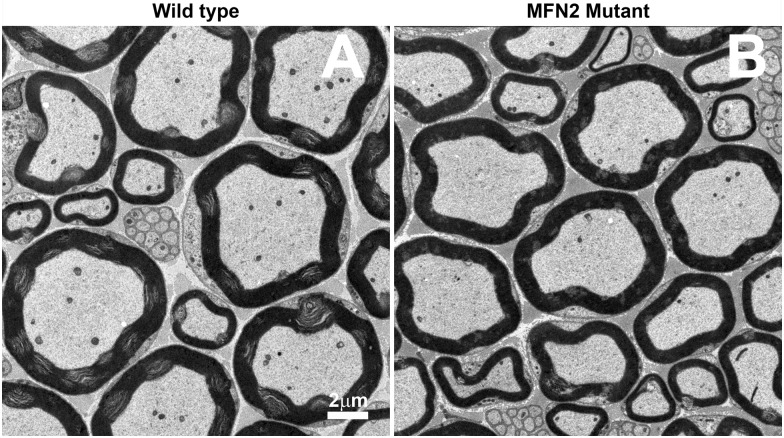
TEM micrographs of wild-type versus nestin MFN2 mutant mice. TEM of transverse sections of control (A) and (B) MFN2 mutant mice tibial nerve showing fewer mitochondria in mutant versus control axons. (scale bar = 2μm).

The number of mitochondrial profiles per axon was 2.67 ± 0.24 vs 1.84 ± 0.40, in control versus MFN2 mutant mice respectively (mean ± stdev, n = 3, p< 0.012 student’s t-test), see Fig 8. Compared to homozygous Rosa-STOP-MFN2^T105M^/CAG-CreER^T2^ (Fig 5), we observed neither abnormal myelination nor mitochondrial aggregation in heterozygote nestin-cre-STOP-MFN2^T105M^ mice.

### The number of myelinating axons, their diameters and degree of myelination (g-ratios) in tibial nerves in nestin-Cre-MFN2 mice were not significantly different from those in age-matched wild-type mice

The number of myelinated axons in the tibial nerves of WT mice was 2299 ± 154 versus 2217 ± 56 in MFN2 heterozygote mutant mice (mean ± std dev, n = 3, difference not significant, (NS)). The mean axonal diameter of myelinated axons in the tibial nerve of WT versus MFN2 mutant mice was 3.63 ± 0.25 microns (mean ± std dev) versus 3.56 ± 0.32 microns respectively (n = 3) (NS). The G-ratio of WT axons was 0.658 ± 0.011 versus 0.662 ± 0.013 mean ± std dev, n = 3) in MFN2 mutant heterozygotes (NS).

### Perturbations in calf skeletal muscles of nestin-Cre-MFN2 mice

**Fig 9:**
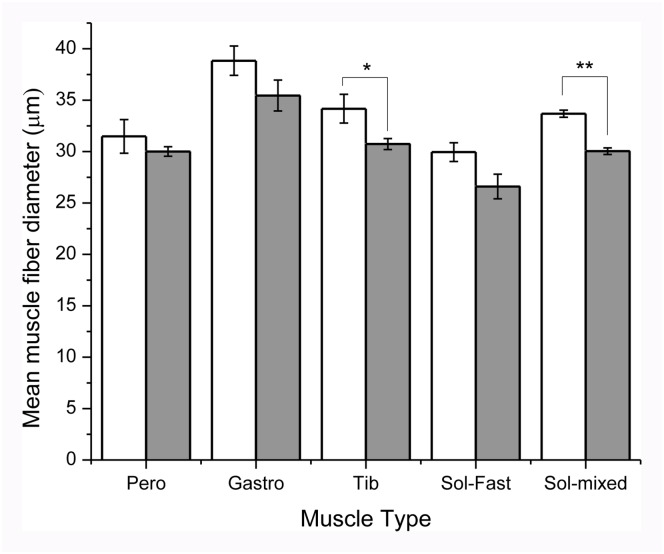
Quantification of muscle fiber diameter in control versus MFN2 mutant mice (see Table 2). Bar histograms quantifying the diameters of fast muscle fibers in the peroneus (Pero), lateral gastrocnemius (Gastro), tibialis anterior (Tib) muscles and the number of fast and slow/mixed fibers in the soleus (Sol) muscle of control (open bars) and MFN2 mutant mice (dark bars) showing mean ± SEM. (* denotes statistical difference, p< 0.03 while ** denotes p< 0.015, n = 4, using Wilcoxon-Mann-Whitney test).

**Fig 10:**
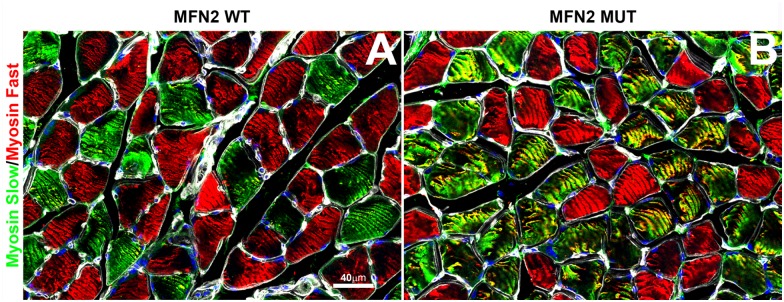
Triple label immunofluorescence labeling of fast/slow myosin isoforms in control versus nestin MFN2 mutant mice. A 0.5μm confocal optical slice of transverse frozen sections of A) Wild-type and B) MFN2 mutant soleus muscles. Note the clear demarcation of fast (red) versus slow (green) myosin ATPase isozymes in A versus the mixed slow/fast fibers in B, (yellow). The outer fiber circumference of each fiber is defined by the basement membrane staining using antibodies to type IV collagen (white) Scale bar = 20μm.

Fig 9 shows there was fast muscle fiber atrophy in the anterior tibialis musculature. Atrophy in the soleus muscle was evident only when comparing the slow fiber diameters in wild-type muscle with mixed slow/fast-converted fibers (Fig 10) in the MFN2 mutant mice.

**Fig 11:**
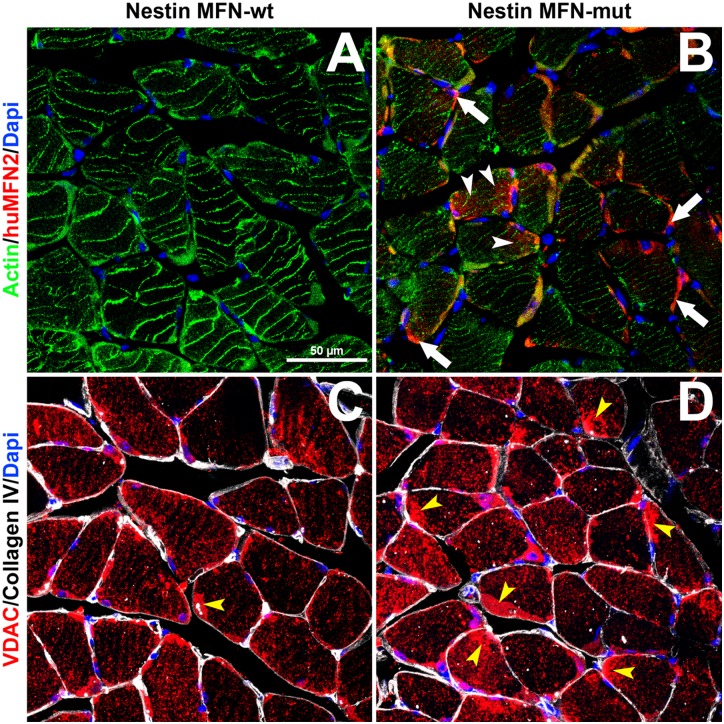
Triple immunofluorescence labeling comparing both sarcomeric actin and VDAC expression in control versus MFN2 mutant mice. A 0.5μm confocal optical slice of transverse frozen sections of A and C wild-type and B and D MFN2 mutant soleus muscle. Micrographs A and B show immunolabelling with sarcomeric actin (green) and the mutated human form of MFN2 (red, seen only in B) and the nuclei of surrounding satellite cells (blue), while C and D demonstrate the distribution of VDAC positive mitochondria (red) with surrounding satellite cell nuclei (blue) encompassed by basement membrane immunolabelling using antibodies to type IV collagen (white). In B, white arrows show the presence of mutant MFN2 expression in satellite cells while arrowheads indicate its diffusion into a myofiber. In C and D, yellow arrowheads show abundant condensed expression of VDAC in satellite cells in the nestin-cre-MFN2 mice versus wild-type mice. In combination, these micrographs other important differences between wild type and MFN2 mutant soleus muscle fibers in addition to fiber atrophy in the latter. Initially, note the selective expression of the mutant form of MFN2 in satellite cells of the mutant myofibers with occasion dissemination into the muscle fiber (white arrow-heads). Also note both the decreased intensity of sarcomeric actin immunolabelling in the mutant mice versus wild-type mice (compare A and B) and the relative disruption of striated myofibrillar distribution of VDAC positive mitochondria in mutant micrograph D versus C. Finally, it is evident that there are many more peripheral satellite cell mitochondria merging with myofibers (yellow arrowheads) in D versus C. Scale bar = 50μm.

We also investigated whether or not soleus muscle fibers and their progenitor pool, namely satellite cells, exhibited other abnormalities, particularly since the latter express nestin [38]. Fig 11 demonstrates a number of perturbations aside from myofiber atrophy when comparing nestin-cre control and heterozygote mutant MFN2 micrographs. Firstly, there was a decrease in sarcomeric actin immunostaining and secondly a disruption in the normal striatal pattern of mitochondrial VDAC expression in nestin MFN2 mutant soleus myofibers versus control fibers. Thirdly, Fig 11D shows evidence of peripheral satellite cell fusion with soleus muscle fibers compared to nestin-cre control mice. On the one hand, it is possible that these perturbations may reflect decreased axonal transport of mitochondria to neuromuscular junctions or alternatively may also reflect the expression of mutant MFN2 by satellite cells.

It is also evident from Figs 10 and 11 that there was an absence of both small angulated myofibers and fiber grouping; typical of neurogenic disorders in general [39].

## Discussion

In this study, we describe for the first time, a clear hind limb motor deficit i.e. decreased hind limb footprint length, in mice hemizygous for expression for the human Thr105Met (T105M) missense mutation in MFN2, a mutation that is common in CMT2A patients. As described in the introduction, Mfn2 is a GTPase that mediates many crucial functions including: fusion of mitochondrial outer membranes; is essential for repair of damaged mitochondria; is responsible for efficient mitochondrial energetics; mitochondrial-endoplasmic reticulum Ca^2+^ coupling. Disruption of mitochondrial distribution and configuration have previously been observed in axons and Schwann cells in CMT2A nerve biopsies.

Our initial studies showed that ‘strong’ hemizygous expression of MFN2^T105M^ in many cell-types (under the control of the CAG-promoter) produced a pathogenic phenotype, requiring mice to be euthanized >6 weeks post tamoxifen induced expression of this allele. This phenotype occurred despite the fact that these mice have 2 normal copies of mouse MFN2. However, our immunohistochemical analysis of wild-type MFN2 expression in control and mutant motor neurons (Fig 2G and 2H) showed that the levels of expression of endogenous MFN2 was decreased in mutant versus wild-type mice. This argues against the possibility that the mutant phenotype was due to overexpression of both forms of MFN2 rather than being due to expression of the mutated form of MFN2.

Given the crucial role of MFN2 in mediating mitochondrial fusion, we anticipated that hemizygous expression of MFN2^T105M^ would result in the accumulation of un-fused mitochondria giving the appearance of mitochondrial aggregation at the light microscopic level (Fig 2). Such aggregation of un-fused mitochondria was demonstrated at the ultrastructural level in Schwann cells. However, the presence of such mitochondrial aggregates was not associated with the abnormal myelinated profiles shown in Fig 5 and therefore was likely due to impaired axonal-Schwann cell interactions.

The cause of the motor deficit in our nestin-Cre-driven mutant MFN2 mice is uncertain. It may involve decreased axonal transport of mitochondria, a finding consistent with the role MFN2 mediates in mitochondrial transport. However, we have yet to directly measure axonal transport or show a proximal/distal alteration in mitochondrial distribution to qualify this supposition. We did not detect a reduction in axonal numbers, size or increase in g-ratio (Fig 7) to account for this phenotype. This finding is in contrast with a previously described study using hemizygous mice with the R94W mutation [29], in which axonal loss and a slight increase in g-ratio were reported in peripheral nerve. Also, in homozygous mice with the R94W mutation, they exhibited increased mitochondrial numbers in distal nerves [28]. These discrepancies may reflect differences in the type of MFN2 mutation, hemizygous/homozygous status or strength of promoter. For instance, the R94W mutation is associated with a more severe neuropathy versus the relatively mild one described in patients with the T105M mutation and/or the fact that the Ella-Cre or neuron specific enolose promoter is more, or less, potent compared to our use of the nestin-cre promoter. Our data clearly shows that the choice of promoter can have profound effects on mutant MFN2 expression as evidenced by the use of the tamoxifen-inducible CAG-Cre-ER^T2^ promoter that causes lethal systemic effects not present in CMT2A patients hemizygous for MFN2 mutations.

We have also identified perturbations in soleus muscle fiber diameter, conversion of slow to mixed slow/fast, decreased sarcomeric actin expression, disruption of striatal mitochondrial organization and indications of increased involvement of satellite cells in maintaining soleus muscle fiber composition (Figs 9 and 10). A recent study inhibiting normal MFN2 expression in muscle fiber preparations clearly indicates that MFN2 plays a role in controlling mitochondrial calcium uptake in skeletal muscle [40]. In particular, the study indicated that MFN2 plays an active role in modulating mitochondrial depolarization/calcium uptake and maintaining the localization of skeletal muscle mitochondria in close apposition to the sarcoplasmic reticulum during muscle contraction. Ultrastructural analysis of soleus muscle fibers would prove useful in determining if mutant MFN2 expression perturbs the tethering of mitochondria to the sarcoplasmic reticulum in our mutant MFN2 paradigm. It is noteworthy that in the hemizygous mutant mice, the expression of human MFN2 was restricted to selective fibers where human MFN2 satellite cells appeared to be in intimate contact with myofibers (Fig 10 and see below). Whether these combined phenotypic changes in skeletal muscle are definitively due to an axonal and/or muscle fiber deficit remains to be determined. This matter, could in part be resolved, by performing nerve conduction velocity studies in both motor and sensory hindlimb nerves (i.e. tibial and sural nerve branches) since these are routinely performed in humans as well as in mice with suspected neuropathies [41].

It is interesting to speculate that the combined atrophy of A) soleus muscle fibers whose contraction elicits plantarflexion of the foot during step down motion and the B) tibialis anterior fibers to mediate dorsiflexion during foot raising, could individually or in combination, contribute to decreased foot print length during active hind limb movements. The presence of ‘slow’ oxidative fibers present in the normal soleus muscle is thought to be a necessary safe guard in preventing fatigue during active hind limb locomotion. Therefore, the conversion of these slow fibers to slow/fast mixed fibers may result in decreased fatigue resistance [42] during plantarflexion of the foot during active catwalk runs. Conversion of slow to mixed slow/fast fibers is indicative of decreased nerve influence (e.g. denervation/disuse atrophy) and increased mechanical loading (e.g. due to muscle atrophy, see [43]). However, as noted above, no loss of axons or perturbations in axonal diameter and myelination were detected in these mice. This supports the possibility that inherent perturbations in muscle function may be involved in heterozygote nestin-Cre-MFN2^T106M^ mutant mice. While sural nerve biopsies in patients with CMT2A are routinely analyzed to identify neurogenic abnormalities, analysis of muscle biopsies are comparatively infrequent, and as far as we are aware this is this first report concerning phenotypic muscle perturbations involving the T105M MFN2 mutation.

In conclusion, we have described perturbations in hind limb/foot gaiting and phenotypic alterations in tibial nerve and calf musculature in mice hemizygous for a pathogenic MFN2 allele. We speculate that the decreased hind limb footprint length present in heterozygote MFN2 mutant mice may result from a combination of potential factors including reduced plantarflexion during downward movements of the foot and decreased dorsiflexion extension during elevation of the foot. Such functional deficits may in turn be caused by one or more of the following observations: 1) reduced numbers of mitochondria in tibial axons, possibly implicating reduced axonal mitochondrial transport as described previously [23–24,26]; 2) atrophy of A) plantarflexion muscle fibers in the soleus and B) dorsiflexion anterior tibialis muscle fibers; 3) fatigue in the soleus muscle due to conversion of slow oxidative fibers to mixed slow/fast myosin fibers; 4) reduced sarcomeric actin expression in soleus myofibers. This study provides a baseline to develop a better understanding of mutant MFN2 pathology and further studies are planned to establish whether or not this pathology results from decreased axonal transport including the potential involvement of more distal tibial nerve branches innervating musculature in the foot as well as investigating sensory nerve involvement in our model of pes cavus using both ultrastructural analysis (e.g. sural nerve) and functional sensory testing (e.g. Von Frey analysis testing foot withdrawal to filaments).
