# Correction: Mice Hemizygous for a Pathogenic Mitofusin-2 Allele Exhibit Hind Limb/Foot Gait Deficits and Phenotypic Perturbations in Nerve and Muscle

**DOI:** 10.1371/journal.pone.0204536

**Published:** 2018-09-18

**Authors:** Peter Bannerman, Travis Burns, Jie Xu, Laird Miers, David Pleasure

The mutant mouse strain name Rosa-STOP-MFN2^T106M^ appears incorrectly throughout the article. The correct allele name should be Rosa-STOP-MFN2^T105M^.
